# The Sustainable Personalised Interventions for Cognition, Care, and Engagement (SPICE) Program: Interim analysis of a twelve week multicomponent intervention for people with dementia and care partners

**DOI:** 10.1002/alz.090597

**Published:** 2025-01-09

**Authors:** Nathan M D'Cunha, Rachael Mitterfellner, Julie Griffin, Lara Wiseman, Stephen Isbel, Kasia Bail, Ian Walker, Sanduni De Silva, Sarah Whitting, Helen Holloway, Sam Kosari, Louise Barrett, Kathleen Rutherford, Michelle Bennett, Diane Gibson

**Affiliations:** ^1^ Centre for Ageing Research and Translation, University of Canberra, Bruce, ACT Australia; ^2^ Canberra Health Services, Bruce, ACT Australia; ^3^ Synergy Nursing and Midwifery Research Centre, Canberra, ACT Australia

## Abstract

**Background:**

The Sustainable Personalised Interventions for Cognition, Care, and Engagement (SPICE) program was developed to address an identified gap in access to high‐quality integrated post‐diagnostic rehabilitation for people with dementia and their care partners in Canberra, Australia. The multicomponent intervention aims to maximise quality of life (QoL) and independence of people with dementia by increasing engagement in everyday and meaningful activities and promoting care partners' physical and mental well‐being.

**Method:**

The SPICE program is a waiting‐list study design delivered by a multidisciplinary allied health team over twelve weeks. The five components are: 1) cognitive stimulation therapy; 2) carer support and capacity building; 3) physical activity; 4) Care of People with dementia in their Environments (COPE) program; and 5) dietary assessment and advice. Each group comprises up to seven people with dementia and their care partners. They engage in group activities twice weekly and receive the COPE program and dietary assessment and advice as a dyad. Outcome measures include QoL, neuropsychiatric symptoms, cognitive and physical function, and carer well‐being. Early feasibility of the program contributed to expansion of the study. Here, we report on the first six completed groups.

**Result:**

Thirty‐eight people with dementia and 38 care partners attended group activities with 92% and 91% attendance, respectively. Participants completed 98% of COPE program appointments and 93% of dietary appointments. Pre‐ to post‐program improvements were observed in proxy‐reported QoL (p = 0.02), reduced neuropsychiatric symptoms (p<0.001) and carer distress related to these symptoms (p = 0.04), and reduced caregiving challenges (p<0.001). Both people with dementia and care partners improved in the Alternating Step Test (AST) (both; p<0.001), as did care partners in Timed Up and Go (TUG) (p = 0.04). Twenty‐six people with dementia and 26 care partners have completed follow‐up, improving from baseline to 24 weeks in self‐reported QoL of the person with dementia (p = 0.03), and reduced neuropsychiatric symptoms (p<0.01) and carer distress related to these symptoms (p = 0.02). Care partners maintained improvements in the AST (p<0.01) and TUG (p = 0.05).

**Conclusion:**

Interim analysis suggests the SPICE program benefits people with dementia and their care partners. Further results are required to support continuation and expansion of the program.

